# A comparative evaluation of sequence classification programs

**DOI:** 10.1186/1471-2105-13-92

**Published:** 2012-05-10

**Authors:** Adam L Bazinet, Michael P Cummings

**Affiliations:** 1 Laboratory of Molecular Evolution, Center for Bioinformatics and Computational Biology, University of Maryland, College Park, MD 20874, USA

## Abstract

**Background:**

A fundamental problem in modern genomics is to taxonomically or functionally classify DNA sequence fragments derived from environmental sampling (i.e., metagenomics). Several different methods have been proposed for doing this effectively and efficiently, and many have been implemented in software. In addition to varying their basic algorithmic approach to classification, some methods screen sequence reads for ’barcoding genes’ like 16S rRNA, or various types of protein-coding genes. Due to the sheer number and complexity of methods, it can be difficult for a researcher to choose one that is well-suited for a particular analysis.

**Results:**

We divided the very large number of programs that have been released in recent years for solving the sequence classification problem into three main categories based on the general algorithm they use to compare a query sequence against a database of sequences. We also evaluated the performance of the leading programs in each category on data sets whose taxonomic and functional composition is known.

**Conclusions:**

We found significant variability in classification accuracy, precision, and resource consumption of sequence classification programs when used to analyze various metagenomics data sets. However, we observe some general trends and patterns that will be useful to researchers who use sequence classification programs.

## Background

A fundamental problem in modern genomics is to taxonomically or functionally classify DNA sequences derived from environmental sampling (i.e., metagenomics). Many metagenomic studies are essentially community ecology studies, which seek to characterize communities statically or dynamically in terms of composition, structure, abundance, demography, or succession, and sometimes with consideration of other biotic or abiotic factors. Consequently many of these characterizations, and inferences derived from them, are sensitive to the accuracy and precision of taxonomic assignment of the metagenomic sequences involved. These sequences are often in the form of unassembled reads whose average length in a sample may vary by an order of magnitude depending on the sequencing technology used (e.g., ∼100 bp to ∼1000 bp). To classify these sequences of unknown origin, the basic strategy employed is to compare them to annotated sequences that reside in public databases (e.g., GenBank [1], Pfam [2]). On the basis of such comparisons, one may be able to say with some certainty that a particular sequence belongs to a specific taxon (of any taxonomic rank from domain to species; more specific classifications are usually more desirable). Sometimes the query sequence does not have a close relative in the database, which is problematic for all methods.

The classification of unlabeled sequences using previously labeled sequences is *supervised* learning; this approach is the focus of our evaluation. However, it is important to mention that *unsupervised* learning techniques exist for “binning” sequences in an environmental sample (e.g., LikelyBin [3], CompostBin [4]), i.e., clustering groups of similar sequences together. These techniques are useful when one desires a high-level characterization of their sample (e.g., classification of bacteria at the phylum rank). Binning may also be used to improve subsequent supervised classification of groups of sequences (PhyScimm [5]).

It is important to note that some supervised learning methods will only classify sequences that contain “marker genes”. Marker genes are ideally present in all organisms, and have a relatively high mutation rate that produces significant variation between species. The use of marker genes to classify organisms is commonly known as DNA barcoding. The 16S rRNA gene has been used to greatest effect for this purpose in the microbial world (green genes [6], RDP [7]). For animals, the mitochondrial COI gene is popular [8], and for plants the chloroplast genes *rbc*L and *mat*K have been used [9]. Other strategies have been proposed, such as the use of protein-coding genes that are universal, occur only once per genome (as opposed to 16S rRNA genes that can vary in copy number), and are rarely horizontally transferred [10]. Marker gene databases and their constitutive multiple alignments and phylogenies are usually carefully curated, so taxonomic and functional assignments based on marker genes are likely to show gains in both accuracy and speed over methods that analyze input sequences less discriminately. However, if the sequencing was not specially targeted [11], reads that contain marker genes may only account for a small percentage of a metagenomic sample.

### General approaches to sequence classification

We have identified three main supervised learning approaches that compare query sequences to database sequences for the purpose of assigning a taxon label: sequence similarity search (homology or alignment-based methods; e.g., BLAST [12]), sequence composition methods (e.g., Markov models, k-mer counts), and phylogenetic methods (which apply an evolutionary model to the query and database sequences and determine where the query best “fits” in the phylogeny). Most software programs use only one of these approaches, but some use a combination of two approaches. (None of the programs mentioned in this paper combine all three approaches).

Programs that primarily utilize sequence similarity search include CARMA [13,14], FACS [15], jMOTU/Taxonerator [16], MARTA [17], MEGAN [18], MetaPhyler [19], MG-RAST [20], MTR [21], and SOrt-ITEMS [22]. Most of these programs employ BLAST (most commonly, BLASTX), and several incorporate some version of the lowest-common ancestor (LCA) algorithm first pioneered by MEGAN. After BLAST, the second most common method aligns a query sequence to a reference sequence represented by a profile hidden Markov model (pHMM); usually a Pfam domain. Alignment-based methods display great accuracy, even for short query sequences, but suffer from two general shortcomings: a) since the reference databases are very large, it can take a long time to search each query sequence against them; and b), if the query sequence is not represented in the database, as could often be the case, assignment accuracy may suffer more so than when using other methods.

Programs that primarily utilize sequence composition models include Naive Bayes Classifier (NBC) [23,24], PhyloPythia [25,26], PhymmBL [27], RAIphy [28], RDP [29], Scimm [5], SPHINX [30], and TACOA [31]. Methods for building sequence models often make use of interpolated Markov models (IMMs), naive Bayesian classifiers, and k-means/k-nearest-neighbor algorithms. There is some overhead to computing sequence models of various organisms, but once models are built, query sequence classification is generally faster than with alignment-based methods. Accuracy, however, may still be able to be improved — this is why PhymmBL incorporates similarity search (the “BL” is for BLAST). As a result, PhymmBL achieves greater accuracy than either Phymm or BLAST alone. Finally, it was widely reported that the initial version of PhyloPythia performed poorly for query sequences less than 1000 bp in length [27,28]; few current next-generation sequencing (NGS) technologies produce reads of that length. However, composition-based methods are now perfectly capable of classifying short query sequences. For example, NBC obtained over 90% accuracy for 25 bp reads with 5-fold cross-validation [23].

Programs that primarily utilize phylogenetic methods include EPA [32], FastTree [33], and pplacer [34]. Phylogenetic methods attempt to “place” a query sequence on a phylogenetic tree according to a model of evolution using maximum likelihood (ML), Bayesian methods, or other methods such as neighbor-joining (NJ). Some programs compute the length of the inserted branch, which represents the amount the query sequence has evolved relative to the rest of the tree; most programs, however, are simply concerned with the placement (and hence classification) of the sequence. Programs assign a specific taxon (and hence taxonomic rank) to a “placed” sequence using different algorithms, but they all make use of the basic observation that an inserted branch will be divergent from an internal node representing a species or higher rank. Since phylogenetic methods require a multiple alignment, and a fixed topology (either derived from the multiple alignment, or some other source; e.g., the NCBI taxonomy), the first step in most phylogenetic workflows is to add a query sequence containing a marker gene to a reference alignment (AMPHORA [35,36], Treephyler [37], green genes [6]). Hence, most phylogenetic methods require the use of marker genes. One that does not, however, is SAP [38], in which the first step is to construct a multiple alignment from the results of a BLAST search. Phylogenetic methods assume that using computationally intensive evolutionary models will produce gains in accuracy, and their inherent use of tree-based data structures makes taxon assignment to higher ranks as well as lower ones very straightforward. The additional algorithmic complexity means that phylogenetic workflows currently require substantial computing power to analyze large metagenomic samples, however; this is true even for methods that only use marker genes. Large-scale analyses will gradually become more practical as more efficient algorithms are developed, computational resources become more powerful, and through use of parallelization.

#### Additional considerations

One important consideration for any sequence classification method is whether the method offers a measure of assignment confidence. Such an uncertainty measure is extremely useful; assignments whose confidence score is below a certain threshold can be disregarded, for example. Phylogenetic methods tend to provide confidence of assignment through use of bootstrap or posterior probabilities, or other techniques. Alignment-based methods generally do not provide a confidence estimate.

Another consideration is the availability and ease of use of the program — whether it is a command line program, has a graphical user interface (GUI), is available as a web service, and so on. If the program is to be downloaded and installed, one must consider how much processing power, memory, and disk the program will need to analyze a particular data set. Some of these needs will prohibit local execution of the program for large data sets, perhaps instead necessitating use of a compute cluster. If there is a web service available for the program, one needs to find out how much computational power is allocated to a single user, and thus whether the service can be used in practice to analyze entire metagenomes. A further consideration is whether the program continues to be actively developed and maintained after a paper is published and the code is initially released. Actively maintained programs are likely to be improved as a result of feedback from users, and may eventually become “standard” tools used by the community.

### Program capability analysis

We identified 25 programs for sequence classification that fall into one of the three primary analysis categories we described: sequence similarity or alignment-based (9 programs), sequence composition model-based (8 programs), and phylogenetic-based (8 programs). Our list is not exhaustive, but we do include a broad cross section of widely used and interesting programs in our comparison.

**Figure 1:**
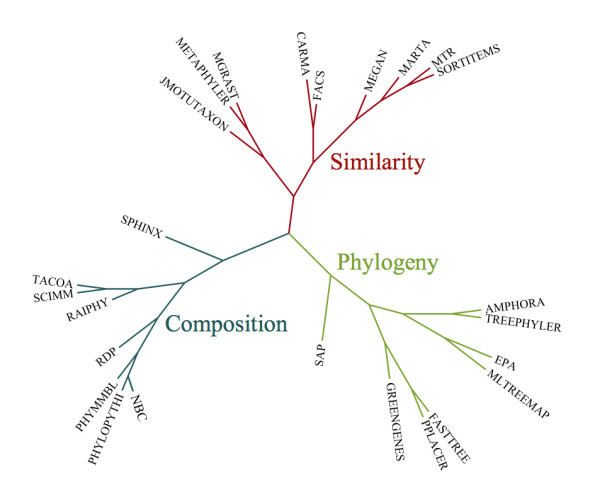
**Program clustering.** A neighbor-joining tree that clusters the classification programs based on their similar attributes.

The attributes and capabilities of each program are given in Table 1. For each program, we report the general analysis method it uses, and more detailed analysis characteristics, as applicable; whether the program requires specific genes as input; and the type of interface to the program. For a given program attribute (a column in Table 1), it is possible for a program to have multiple values. We defined a distance function and created a neighbor-joining tree that clusters the programs based on their similar attributes (Figure 1).

**Table 1:** Program attributes and characteristics

***Similarity-based Methods***					
**Program**	**Similarity Method**	**LCA**	**Specific Genes Req’d**	**Interface**	
CARMA	BLAST, HMM				command line, web-based
FACS	other				command line
jMOTU/Taxonerator	BLAST, other			multiple alignment	command line
MARTA	BLAST	LCA-like		command line	
MEGAN	BLAST	LCA-like		GUI	
MetaPhyler	BLAST			marker genes	command line
MG-RAST	BLAST			marker genes	web-based
MTR	BLAST	LCA-like		command line	
SOrt-ITEMS	BLAST	LCA-like		command line	
***Composition-based Methods***					
**Program**	**Composition Method**	**Machine Learning**	**Confidence Method**	**Specific Genes Req’d**	**Interface**
Naive Bayes Classifier	NBC	supervised	other		command line, web-based
PhyloPythiaS	other	supervised			command line, web-based
PhymmBL	IMM	supervised	other		command line
RAIphy	other	semi-supervised			GUI
RDP	k-means/kNN, NBC	supervised	bootstrap	16S rRNA	command line, web-based
Scimm	IMM	semi-supervised			command line
TACOA	k-means/kNN	supervised			command line
***Phylogeny-based Methods***					
**Program**	**Phylogeny Method**	**Confidence Method**	**Specific Genes Req’d**	**Interface**	
EPA	ML	bootstrap, other	multiple alignment	command line, web-based	
FastTree	other	bootstrap	multiple alignment	command line	
green genes (NAST, Simrank)	other			16S rRNA	web-based
pplacer	ML, Bayesian	posterior probability, other	multiple alignment	command line	
***Combined Similarity and Composition-based Methods***					
**Program**	**Similarity Method**	**Composition Method**	**Machine Learning**	**Specific Genes Req’d**	**Interface**
SPHINX	BLAST	k-means/kNN	supervised		web-based
***Combined Similarity and Phylogeny-based Methods***					
**Program**	**Similarity Method**	**Phylogeny Method**	**Confidence Method**	**Specific Genes Req’d**	**Interface**
AMPHORA	HMM	other	bootstrap	marker genes	command line
MLTreeMap	BLAST, HMM	ML	bootstrap, other	marker genes	command line, web-based
SAP	BLAST	Bayesian, other	posterior probability, other		command line
Treephyler	HMM	other	bootstrap	marker genes	command line

### Program performance evaluation

When publishing their method, researchers typically compare their program to one or more existing programs. Presumably they attempt to choose programs that are most similar to their own, but we find that this is not always the case. Perhaps the researcher is simply not aware of all the tools in existence, or does not have the time to evaluate them all, so they pick a couple of popular or well-known tools. In contrast, we focused our comparisons on a single category at a time, which we believe generates more interesting and generally useful comparisons between conceptually similar programs.

We evaluated the performance of sequence classification programs in two main areas: 

1. *assignment accuracy* — we tested assignment accuracy using data sets from the publications associated with each program, and analyzed each data set with as many programs from the corresponding category as possible. Specifically, we measured assignment sensitivity (*number of correct assignments* / *number of sequences in the data set*), precision (*number of correct assignments* / *number of assignments made*), the overall fraction of reads that were assigned, and the taxonomic rank at which assignments were made. (In general, more specific taxon assignments are more useful, although one usually expects sensitivity and precision to decrease as increasingly specific assignments are made).

2. *resource requirements* (processing time, RAM, and disk requirements) — we monitored the resources consumed by each program during the analysis of each data set. Some programs have web services available that we used in program evaluation, which made it more difficult to precisely measure how much of each resource was consumed.

## Results

Within each category, we selected a subset of programs to evaluate. Programs were selected on the basis of several factors: whether they are actively maintained, how popular they are, how recently they were published, whether they have been superseded by another program, and so on. From this standpoint, we have attempted to make the comparisons in each category as interesting and useful to the current active community of researchers as possible.

### Alignment

In the alignment category, we selected five programs to evaluate: CARMA (command line version 3.0), FACS (1.0), MEGAN (4.61.5), MG-RAST (3.0), and MetaPhyler (1.13). Based on our experience using these programs, we note the following: 

1. FACS requires bloom filters to be built for the reference sequences that are to be searched, which is infeasible to do for large databases (e.g., GenBank’s non-redundant nucleotide (nt) and protein (nr) databases). Therefore, we were unable to analyze the majority of data sets with FACS.

2. We ran BLASTX with default parameters against the nr database, and used this as input to CARMA and MEGAN. BLAST accounted for 96.40% and 99.97% of the total runtime for these workflows, respectively (Table 2).

3. MG-RAST has several different analysis options. We used the non-redundant multi-source annotation database, or M5NR, and their implementation of an LCA algorithm for taxon assignment.

4. MG-RAST requires input sequences to contain protein-encoding genes (PEGs), and assigns each of these to a particular taxon. Not all query sequences in a random shotgun sample will contain a PEG, so MG-RAST typically classifies fewer overall sequences than other methods. In addition, it is possible for a single input sequence read to contain multiple PEGs. In order to be consistent with other methods that make classifications on a read-by-read basis, we map the PEG assignments back the read they came from, and make fractional read assignments to a particular taxon as necessary. (For example, a particular read could contain two PEGs: one PEG assigned to phylum A, and the other PEG assigned to phylum B. If only one of these is correct, the read would contribute 0.5 to a tally of “correct” assignments, and 0.5 to a tally of “incorrect” assignments).

5. MetaPhyler requires input sequences to contain certain “marker genes” (protein-coding genes that are “universal” and occur only once per genome), an approach pioneered by AMPHORA. Very few query sequences in a random shotgun sample will contain marker genes, so MetaPhyler typically classifies fewer overall sequences than other methods; many fewer than even MG-RAST, for example.

**Table 2:** Performance of alignment-based programs

**Program**	**FACS 269 bp**	**MetaPhyler 300 bp**	**CARMA 265 bp**	**PhyloPythia 961 bp**	**Mean**
**Percentage of sequence classified**					
CARMA	29.0	93.6	68.7	61.3	63.2
MEGAN	48.4	88.2	90.5	62.2	72.3
MetaPhyler	0.2	80.9	0.5	0.6	20.6
MG-RAST	27.1	29.8	80.2	70.5	51.9
**Sensitivity (percentage)**					
CARMA	26.7	93.4	68.5	59.8	62.1
MEGAN	42.5	87.9	90.3	61.0	70.4
MetaPhyler	0.1	80.7	0.5	0.5	20.5
MG-RAST	25.0	29.7	80.1	67.2	50.5
**Precision (percentage)**					
CARMA	92.0	99.7	99.7	97.4	97.2
MEGAN	78.1	99.7	99.8	98.1	93.9
MetaPhyler	84.0	99.7	100.0	83.8	91.9
MG-RAST	92.4	99.8	99.9	95.3	96.9
**CPU Runtime (minutes)**					
CARMA^1,2^	290880	77340	74950	360107	200819
MEGAN^1,2^	288020	72060	72010	351060	195788
MetaPhyler^3^	10	20	2	28	15
MG-RAST^4^	60	10080	20160	12960	10815
**Memory Usage (Megabytes of RAM)**					
CARMA	100	100	100	120	105
MEGAN	1024	1024	1024	1410	1121
MetaPhyler	5734	5734	5734	5734	5734
MG-RAST^5^	-	-	-	-	-

Measurements of sensitivity, precision, and resource consumption on four simulated data sets.

^1^analysis performed on a 2.66 GHz Intel Core i7 MacBook Pro running Mac OS X 10.7.1 with 8 GB 1067 MHz DDR3 RAM.

^2^BLAST v2.2.18 analysis performed using ∼200 Opteron 2425 HE (2.1GHz) cores; each node has 48G RAM.

^3^analysis performed on an AMD Opteron 250 (2.4 GHz) Sun Fire V40z with 32 GB RAM.

^4^used web service; recorded value is number of minutes to receive results, not actual CPU runtime.

^5^used web service; memory usage was unable to be determined.

Four data sets were selected for analysis with each of the alignment-based programs. Percentage of sequence classified, sensitivity, precision, and resource consumption are shown for the alignment-based programs in Table 2. What follows is a short description of each data set, and a summary of the results of analysis with each program.

#### FACS 269 bp high complexity 454 metagenomic data set

This data set, which consists of 1^05^sequences of average length 269 bp, originally used by Stranneheim et al. [15], was downloaded from the FACS web site. The sequences are from 19 bacterial genomes, three viral genomes, and two human chromosomes. The distribution of sequences is as follows: 73.0% Eukaryota, 25.6% bacteria, and 1.5% viruses.

It was reported that FACS assigned sequences to species with 99.8% sensitivity and 100% specificity using a k-mer size of 21 and a match cutoff of 35% sequence similarity [15]. However, we encountered technical difficulties using the FACS software and were unable to reproduce the results reported in the FACS paper.

Distribution of sequence assignments produced by the alignment-based programs is shown in Table 3.

**Table 3:** Results for the FACS simHC metagenomic data set (1^05^sequences, 269 bp)

	**actual**	**CARMA**	**MEGAN**	**MetaPhyler**	**MG-RAST**	
percentage of sequence classified		29.0	54.4	0.2	27.1	
Eukaryota	73.0	30.3	42.0	0.0	21.0	
Bacteria	25.6	62.8	52.0	84.0	71.5	
Viruses	1.5	0.0	0.3	0.0	0.1	
Archaea	0.0	6.9	5.7	16.0	7.3	
percentage of sequence misclassified		8.0	12.2	16.0	7.6	
correlation coefficient		0.45	0.72	-0.09	0.26	

The actual distribution of sequences compared to the distribution inferred by the alignment-based programs.

#### MetaPhyler 300 bp simulated metagenomic data set

This data set, which consists of 73,086 sequences of length 300 bp, originally used by Liu et al. [19], was acquired from the authors. The sequences are simulated reads from 31 phylogenetic marker genes from bacterial genomes. The distribution of sequences into bacterial phyla is as follows: Proteobacteria, 47.0%; Firmicutes, 21.9%; Actinobacteria, 9.7%; Bacteroidetes, 4.8%; Cyanobacteria, 3.9%; Tenericutes, 2.2%; Spirochaetes, 1.9%; Chlamydiae, 1.3%; Thermotogae, 0.9%; Chlorobi, 0.9%.

Although a comparison of MetaPhyler, MEGAN, CARMA, and PhymmBL is already given for this data set [19], we decided to redo these analyses in a way that is consistent with our standard procedures (i.e., we did not exclude query reads from the reference database, as Liu et al. did with 3/4 of their analyses, viz., MetaPhyler, MEGAN, and PhymmBL). Additionally, we restricted our analyses to the phylum rank.

Distribution of sequence assignments produced by the alignment-based programs is shown in Table 4.

**Table 4:** Results for the MetaPhyler simulated metagenomic data set (73,086 sequences, 300 bp)

	**actual**	**CARMA**	**MEGAN**	**MetaPhyler**	**MG-RAST**	
percentage of sequence classified		93.6	88.2	80.9	29.8	
Proteobacteria	47.0	47.6	44.5	48.3	46.7	
Firmicutes	21.9	22.2	24.0	21.8	23.1	
Actinobacteria	9.7	8.7	8.8	9.1	9.3	
Bacteroidetes	4.8	4.5	4.8	4.3	4.4	
Cyanobacteria	3.9	3.6	3.8	3.9	3.7	
Tenericutes	2.2	2.5	2.7	2.4	2.3	
Spirochaetes	1.9	2.4	2.6	2.3	2.2	
Chlamydiae	1.3	1.9	2.0	1.8	1.8	
Thermotogae	0.9	1.2	1.2	1.1	1.2	
Chlorobi	0.9	1.4	1.5	1.3	1.4	
percentage of sequence misclassified		0.3	0.3	0.3	0.2	
correlation coefficient		≈ 1.0	≈ 1.0	≈ 1.0	≈ 1.0	

The actual distribution of sequences compared to the distribution inferred by the alignment-based programs.

#### CARMA 265 bp simulated 454 metagenomic data set

This data set, which consists of 25,000 sequences of average length 265 bp, originally used by Gerlach and Stoye [14], was acquired from the WebCARMA web site. The sequences are simulated 454 reads from 25 bacterial genomes. The distribution of sequences into bacterial phyla is as follows: Proteobacteria, 73.0%; Firmicutes, 12.9%; Cyanobacteria, 7.8%; Actinobacteria, 5.2%; Chlamydiae, 1.0%.

Distribution of sequence assignments produced by the alignment-based programs is shown in Table 5.

**Table 5:** Results for the CARMA 454 simulated metagenomic data set (25,000 sequences, 265 bp)

	**actual**	**CARMA**	**MEGAN**	**MetaPhyler**	**MG-RAST**	
percentage of sequence classified		68.7	90.5	0.5	80.2	
Proteobacteria	73.0	73.2	73.0	69.2	73.2	
Firmicutes	12.9	13.2	12.8	17.3	12.9	
Cyanobacteria	7.8	7.3	7.8	6.8	7.6	
Actinobacteria	5.2	5.0	5.3	2.3	5.4	
Chlamydiae	1.0	1.2	1.1	4.5	0.9	
percentage of sequence misclassified		0.3	0.2	0.0	0.1	
correlation coefficient		≈ 1.0	≈ 1.0	≈ 1.0	≈ 1.0	

The actual distribution of sequences compared to the distribution inferred by the alignment-based programs.

#### PhyloPythia 961 bp simMC data set

This data set, which consists of 124,941 sequences of average length 961 bp, originally used by Patil et al. [39], was downloaded from the FAMES [40] web site. All classifications were performed at the genus rank.

#### Discussion

From the alignment-based analyses, we can make several observations. 

1. The BLAST step completely dominates the runtime for alignment-based methods. It can use a fair amount of disk space in the process (as much as 17 GB for the MetaPhyler data set), and can use a considerable amount of RAM if analyzing a large number of sequences on a single node.

2. MetaPhyler is the one exception to the previous observation; its BLAST step and subsequent algorithmic steps run extremely quickly, but it generally only classifies a small fraction of reads in a typical sample. Also, Table 2 shows that MetaPhyler uses a large amount of RAM (5.6 GB); this is in part due to a memory leak that has been fixed in a subsequent release (personal correspondence with the author).

3. The MG-RAST web service showed a large variance in time required to receive results, although there is at least a weak correlation with data set size and analysis parameters. With a web service, it is difficult to know what other variables affect time to results (e.g., load on cluster queues), and currently the MG-RAST server does not provide an estimate of how long a given submission will take.

4. For the FACS high complexity data set, none of the programs produced a taxonomic distribution that was remotely close to the known distribution (Table 3); all greatly underestimated the amount of eukaryotic DNA. The reason for this is unclear.

5. For the MetaPhyler 300 bp data set, all four alignment programs recapitulated the known distribution of bacterial phyla extremely well (Table 4). All had near-perfect precision, and sensitivity was greater than 80% for 3/4 of the programs (Table 2). MG-RAST only had sensitivity of 30%, but this was still enough assignments to accurately estimate the taxonomic distribution (Pearson’s *r*≈ 1).

6. For the CARMA 265 bp data set, CARMA, MEGAN, and MG-RAST recapitulated the known distribution of bacterial phyla extremely well (Table 5). MetaPhyler was slightly worse, but still quite good considering that it only classified 0.5% of sequences.

7. For the PhyloPythia 961 bp data set, all programs except MetaPhyler displayed comparable sensitivity and precision (Table 2).

8. Methods that use marker genes (MetaPhyler and MG-RAST) are generally less sensitive than methods that do not use marker genes (CARMA and MEGAN), but typically run faster (Table 2). All methods displayed comparable overall precision; CARMA and MG-RAST were the most precise (Table 2).

### Composition

In the composition category, we selected four programs to evaluate: Naive Bayes Classifier (version 1.1), PhyloPythiaS (1.1), PhymmBL (3.2), and RAIphy (1.0.0). Based on our experience using these programs, we note the following: 

1. All four programs need to be “trained” (classifiers built on training data) before they can be used to classify unknown query sequences. Training times for all four programs can be found in Table 6.

2. NBC, PhyloPythiaS, and PhymmBL were all trained on the latest microbial genomes in the RefSeq [41] database.

3. The database we used for RAIphy is the one currently available on the RAIphy web site, which was built from RefSeq in 2010. We built our own database using the latest version of RefSeq and retrained RAIphy with this updated database, but found that classification accuracy was drastically lower. We have been in contact with the developers about the problem, but so far no satisfactory explanation has been found.

4. Technical limitations having to do with memory usage or program bugs required us to break up our FASTA input files into multiple, smaller input files to use with PhyloPythiaS and PhymmBL.

5. NBC produces raw output as hundreds of large matrices, in which the rows represent genomes and the columns represent sequence reads. The value in a particular cell is the score given by the algorithm for assigning a particular sequence read to a particular genome. Therefore, it was necessary to parse this output to find the largest score in each column in order to assign each read to a particular taxon.

**Table 6:** Performance of composition-based programs

**Program**	**PhyloPythia 961 bp**	**PhymmBL 243 bp**	**RAIphy 238 bp**	**Mean**	**Training**
**Percentage of sequence classified**					
NBC	100	100	100	100	
PhyloPythiaS	3.5	3.1	3.3	3.3	
PhymmBL	100	99.7	100	99.9	
RAIphy	100	100	100	100	
**Sensitivity (percentage)**					
NBC	95.4	97.5	99.4	97.4	
PhyloPythiaS	3.1	1.8	2.2	2.4	
PhymmBL	48.4	96.8	81.9	75.7	
RAIphy	54.8	31.8	48.0	44.9	
**Precision (percentage)**					
NBC	95.4	97.5	99.4	97.4	
PhyloPythiaS	88.1	58.5	66.1	70.9	
PhymmBL	48.4	97.0	81.9	75.8	
RAIphy	54.8	31.8	48.0	44.9	
**CPU Runtime (minutes)**					
NBC^1^	13496	3595	17573	11555	1217
PhyloPythiaS^2^	297	180	506	328	4320
PhymmBL^1^	15600	1035	23508	13381	2880
RAIphy^3^	105	25	122	84	30
**Memory Usage (Megabytes of RAM)**					
NBC	200	200	200	200	
PhyloPythiaS^4^	100	100	100	100	
PhymmBL^4^	100	100	100	100	
RAIphy	500	335	400	412	

Measurements of sensitivity, precision, and resource consumption on three simulated data sets.

^1^analysis performed on an AMD Opteron 250 (2.4 GHz) Sun Fire V40z with 32 GB RAM.

^2^analysis perfomed on an AMD Opteron 248 (2.2 GHz) workstation with 8 GB RAM.

^3^analysis performed on a 2.66 GHz Intel Core i7 MacBook Pro running Mac OS X 10.7.1 with 8 GB 1067 MHz DDR3 RAM.

^4^input sequences were broken up into smaller files.

Three data sets were selected for analysis with each of the composition-based programs. Percentage of sequence classified, sensitivity, precision, and resource consumption are shown for the composition-based programs in Table 6. What follows is a short description of each data set, and a summary of the results of analysis with each program.

#### PhyloPythia 961 bp simMC data set

This data set, which consists of 124,941 sequences of average length 961 bp, originally used by Patil et al. [39], was downloaded from the FAMES [40] web site. All classifications were performed at the genus rank.

#### PhymmBL 243 bp RefSeq data set

This data set, which consists of 80,215 sequences of average length 243 bp, originally used by Brady and Salzberg [27], was downloaded from the PhymmBL web site. All classifications were performed at the genus rank.

#### RAIphy 238 bp RefSeq data set

This data set, which consists of 477,000 sequences of average length 238 bp, originally used by Nalbantoglu et al. [28], was downloaded from the RAIphy web site. All classifications were performed at the genus rank.

#### Discussion

From the composition-based analyses, we can make several observations. 

1. PhyloPythiaS took the longest to train (∼ 3 days), but its classification step was relatively fast (∼41× faster than PhymmBL). However, the fastest program was RAIphy, which took a negligible amount of time to train, and classified sequences ∼4× faster than PhyloPythiaS and ∼159× faster than PhymmBL (Table 6).

2. NBC displayed the highest average sensitivity and precision (97.4%), and PhymmBL displayed the second-highest average sensitivity and precision (76%) (Table 6).

3. PhyloPythiaS displayed very low average sensitivity (2.4%), but competitive average precision (70.9%) (Table 6).

4. Average precision is lower for composition-based programs than for alignment-based programs, but this is probably mainly due to the fact that classifications were made at the genus rank for composition-based classifications, and primarily at the phylum rank for alignment-based classifications (Tables 2 and 6).

5. Composition-based programs are supposed to excel at classifying sequences that are not exactly represented in the database, so it would be interesting to compare the performance of these programs in that type of analysis (see “clade-level exclusions” in Brady and Salzberg [27]).

### Phylogenetics

In the phylogenetics category, we selected two programs to evaluate: MLTreeMap (version 2.061) and Treephyler (1.1). Based on our experience using these programs, we note the following: 

1. The MLTreeMap web interface limits an analysis to 50,000 sequences, so we used the command line version. The MLTreeMap workflow makes callouts to BLAST, Gblocks [42], HMMER [43], and RAxML [44], and is very sensitive to the versions of these dependencies used, so it is important to use the specific versions of these programs that are bundled with MLTreeMap.

2. Treephyler requires that the input sequences be converted to amino acids, and corresponding UFO [45] assignments provided. Thus, we performed a 6-frame translation of our DNA input sequences, and used the UFO web server to assign protein sequences to Pfam domains. These files were then used as input to Treephyler.

3. Treephyler is capable of utilizing multiple processing cores during analysis.

The only simulated data set associated with the MLTreeMap and Treephyler publications is the simulated medium complexity (simMC) PhyloPythia data set, so we analyzed this with both programs. Percentage of sequence classified, sensitivity, precision, and resource consumption are shown for the phylogenetic-based programs in Table 7.

**Table 7:** Performance of phylogenetic-based programs

**Program**	**% of sequence classified**	**Sensitivity (%)**	**Precision (%)**	**CPU Runtime (minutes)**
MLTreeMap^1^	0.9	0.8	81.4	3344
Treephyler^1^	6.6	6.3	95.7	7444

Measurements of sensitivity, precision, and resource consumption on the PhyloPythia 961 bp data set.

^1^analysis performed on an AMD Opteron 250 (2.4 GHz) Sun Fire V40z with 32 GB RAM.

#### PhyloPythia 961 bp simMC data set

This data set, which consists of 124,941 sequences of average length 961 bp, originally used by Patil et al. [39], was downloaded from the FAMES web site. All classifications were performed at the genus rank.

#### Discussion

From the phylogenetic-based analyses, we can make several observations. 

1. Treephyler took twice as long to run as MLTreeMap, but was ∼8× more sensitive and achieved higher precision. (Table 7).

2. MLTreeMap and Treephyler made some assignments at taxonomic ranks higher than genus that were not included in this analysis, but would otherwise be useful.

3. MLTreeMap and Treephyler are capable of producing measures of confidence of assignment, which we did not include in this analysis but would be of practical use in most scenarios.

### Comparison of all programs

All 10 programs were used to analyze the simulated medium complexity (simMC) PhyloPythia data set, so it is interesting to compare their relative performance on this particular data set. 

1. Composition-based programs displayed the highest average sensitivity (50.4%), and alignment-based programs displayed the highest average precision (93.7%) (Tables 2 and 6).

2. The two most computationally expensive programs, CARMA and MEGAN, achieved the highest precision (97.4% and 98.1%, respectively) (Table 2).

3. In terms of best combined sensitivity and precision, NBC outperformed all other programs, achieving sensitivity and precision of 95.4% (Table 6).

## Conclusions

The performance of a particular category of programs varied substantially between data sets. The precise reasons for this are likely a complex function of sample taxonomic composition and diversity, level of sequence representation in databases, read lengths and read quality. In general, however, if a data set was challenging for one program, it was challenging for the other programs in that category. The overall variance of the statistics makes it difficult to make definitive statements about the superiority of one program or method over another, but we can state some broad conclusions.

In general, high sensitivity is undesirable if corresponding precision is low. However, very precise methods that do not assign a large fraction of sequences may still be useful, depending on the application. For example, we have shown that in some cases, classifying only a small percentage of a sample may still be enough to recapitulate the correct organismal distribution, especially at a high rank (e.g., phylum). Methods that search for marker genes in a metagenomic sample interrogate relatively few sequences, but as a consequence run quickly and with high precision. In a targeted sequencing experiment, phylogenetic methods and other methods that use marker genes might thus be especially appropriate.

In general, composition-based programs classified sequences the fastest, once they were trained. Phylogenetic programs might be the most computationally intensive on a per-read basis, but owing to their use of marker genes only ran for an intermediate amount of time in our experiments. As expected, BLAST-based programs that did not use marker genes consumed the bulk of the computing resources in our study. Researchers should take note of the fact that programs vary by orders of magnitude in computational resource requirements, and should thus choose programs appropriately depending on the computing resources they have access to, the amount of data to analyze, and the particular bioinformatic application. In addition, some programs are much easier to set up and use than others. Of course, there is often a tradeoff between level of flexibility and configurability, and ease of use.

Taxonomic sequence classification is a fundamental step in metagenomic analyses, as classification accuracy has a direct impact on downstream analyses and the conclusions drawn from them. Therefore, it is important to be aware of the wide variety of tools that currently exist to address this need, and to choose the best performing and most appropriate tools for a given analysis and set of resource constraints.

## Methods

### Program classification

Table 1 was created and filled in manually using appropriate literature, program web sites, and documentation as necessary. In order to cluster the programs, we wrote a Perl script to construct a matrix containing a measure of similarity, or distance, for each possible pair of programs, defined as follows:

*distance*(*program*1,*program*2)$\;=\overset{n}{\underset{a=1}{\sum }}\mathrm{distance}(\mathrm{program}1[a],\mathrm{program}2[a])$where *n* is the number of program attributes (equal to the number of columns in the table).Distances are calculated as follows: 

**if** program1[a] == program2[a]**then** distance(program1[a], program2[a]) = 0 **elseif** common(program1[a], program2[a]) == 0 **then** distance(program1[a], program2[a]) = 1 **else***distance*(*program*1[*a*],*program*2[*a*])$=\frac{\mathrm{common}(\mathrm{program}1[a],\mathrm{program}2[a])}{\mathrm{greater}(\mathrm{program}1[a],\mathrm{program}2[a])}$**endif**

where *common*(*program*1[*a*],*program*2[*a*]) = the number of elements the two attributes share in common, and *greater*(*program*1[*a*],*program*2[*a*]) = the number of elements in the attribute with the greater number of elements.

The distance matrix was provided as input to the NEIGHBOR program from the PHYLIP package [46]. The resulting neighbor-joining tree was plotted in FigTree [47] and labeled to produce Figure 1.

### Tool usage and result processing

Custom Perl scripts were written to parse correct annotations out of the FASTA headers of the various input files for each data set. The PhymmBL data files did not contain annotations, so we used NCBI E-Utilities to access the NCBI taxonomy database and retrieve the scientific classification for each sequence. The classifications made by each program were also parsed out of program output files with Perl scripts, and compared to the correct annotations to calculate sensitivity and precision.

Pearson’s correlation coefficient was used to compare the known distribution of bacterial phyla to the classifications made by the various alignment programs via the cor() function in R [48].

Runtimes were calculated in minutes of wall clock time; if a process ran in parallel, then the runtime was multiplied by the number of parallel processes. The runtimes are not directly comparable because analyses used heterogeneous hardware. Memory usage was calculated by inspecting process memory usage intermittently, and thus is also imperfect. Both measures should still serve as the basis for a rough comparison, however.

## Competing interests

The authors declare that they have no competing interests.

## Author’s contributions

The authors jointly conceived of the study and participated in its design and execution. ALB performed the analyses and drafted the manuscript. MPC assisted with revising the manuscript and formatting it for publication. Both authors read and approved the final manuscript.
